# Clinical Features in Juvenile-Onset Ankylosing Spondylitis Patients Carrying Different B27 Subtypes

**DOI:** 10.1155/2015/594878

**Published:** 2015-07-26

**Authors:** Yikun Mou, Pingping Zhang, Qiuxia Li, Zhiming Lin, Zetao Liao, Qiujing Wei, Jieruo Gu

**Affiliations:** Department of Rheumatology, The Third Affiliated Hospital of Sun Yat-Sen University, Guangzhou, Guangdong 510630, China

## Abstract

*Background*. Ankylosing spondylitis (AS) is a common rheumatic disease and is characterized by inflammation of the axial skeleton. HLA-B27 is strongly associated with AS. Juvenile-onset AS (JAS) with disease onset before 16 years of age differs from adult-onset AS (AAS) in many respects. *Objective*. To compare the clinical features in JAS with different B27 subtypes and analyze the differences between JAS and AAS. *Methods*. 145 JAS and 360 AAS patients were included. The demographic data, clinical manifestations, laboratory markers, Bath AS indices, and B27 subtypes were recorded. *Results*. Peripheral arthritis, enthesitis, BASDAI, ESR, and CRP were significantly higher in JAS patients with HLA-B^*^2704 than those with B27-negative. Enthesitis and ESR were significantly higher in patients with HLA-B^*^2705 than those with B27-negative. The onset age of HLA-B^*^2715 group was much earlier than the other groups. The peripheral arthritis, enthesitis, and hip joint involvement in JAS with HLA-B^*^2704 were significantly higher than those in AAS with HLA-B^*^2704. *Conclusion*. JAS with different B27 subtypes had similar features in most of manifestations; JAS and AAS patients with the same subtype could have distinctive courses. Early diagnosis, hip detection, and control of systemic active inflammation in JAS patients will be helpful for improving the prognosis.

## 1. Introduction

Ankylosing spondylitis (AS) is an inflammatory disorder mainly affecting the axial joints and distinguished by a significant association with HLA-B27 [1, 2]. Juvenile-onset AS (JAS), that is, having onset of symptoms before 16 years of age, is the major part of juvenile spondyloarthropathies (JSpA). In China, JAS accounts for 27.8%~29.8% [3, 4]. Because the individual differences in the clinical manifestations are large and sacroiliac joints of children are in the developmental stage, so imaging diagnoses are limited, and diagnosis may be delayed. JAS has different phenotype and prognosis than adult-onset AS (AAS) [5]. Many studies report about clinical features of JAS [3, 6], and little about those in JAS patients carries different B27 subtypes.

We suspected whether there was different pathological mechanism in JAS. In our previous studies, a group of JAS patients have been typed into B27 subtypes; the positive rate of B27 subtypes in JAS group had no statistical difference compaired with AAS group [7]. We further analysed the clinical manifestations of JAS with different B27 subtypes in this study, so as to investigate the clinical and pathological mechanism of JAS.

## 2. Patients and Methods

### 2.1. Patient Population

From June 2005 to April 2013, 145 JAS and 360 AAS outpatients and inpatients registered in rheumatology department of our hospital were included in this study. All the patients fulfilled the modified New York criteria for AS. Patients with the concomitant presence of chronic renal or hepatic disease, blood disorders, endocrine system diseases, and various acute infections or other infectious diseases were excluded.

Informed consent was obtained from each patient involved in the study.

### 2.2. Biochemical Parameters

C reactive protein (CRP) and erythrocyte sedimentation rate (ESR) were measured by standard laboratory methods.

### 2.3. Baseline Disease Activity Measures

The following measures were obtained at baseline visit: Bath ankylosing spondylitis disease activity index (BASDAI) score (0 = none, 10 = worst) and Bath ankylosing spondylitis functional index (BASFI) score (0 = none, 10 = worst).

### 2.4. Joint Counts

In the 44 joints, which include bilateral proximal interphalangeal joints, metacarpophalangeal joints, wrist, elbow, shoulder, acromioclavicular, sternoclavicular joint, knee, ankle joints, and metatarsophalangeal joint, the number of joints with swelling and tenderness was recorded.

### 2.5. Statistical Analysis

Statistical software SPSS 19.0 for Windows was used to analyze data. Normally distributed measurement data was shown as “mean ± standard deviation,” non-normally distributed data as “median (lower quartile-upper quartile).” The original data of BASFI, ESR, and CRP were skewed distribution, respectively, but after logarithmic transformation, the data became normal distribution, and then they were analyzed. The mean values of two independent samples were compared using *t*-test; comparison between multiple groups was analyzed using one-way ANOVA, while pairwise comparisons between groups were analyzed using LSD-*t* test. Chi-square test was used to analyze constituent ratio. Kruskal-Wallis test was used for comparison in multigroups; Mann-Whitney *U* test was used for comparison between two groups. Differences were considered significant at *P* < 0.05.

## 3. Results

### 3.1. Characteristics of the JAS and AAS Patients

The mean age of 145 JAS patients, including 136 males and 9 females (male : female = 15.1 : 1), was 21.3 ± 7.6 years (from 10 to 43 years). Among them, 35 patients were ≤ 16 y and 110 > 16 y. The onset age was 5 to 16 years, and the average onset age was 13.49 ± 2.36 years. The median duration of disease was 6 years (0.04 to 27 years). 104 patients were sporadic and 41 individuals had family histories (sporadic cases: cases with family histories = 2.54). Mean age of the 360 AAS patients (306 male, 54 female) was 30.5 ± 7.6 years, with mean duration of disease 6.9 ± 5.7 years (0.3 to 35 years).

### 3.2. Comparison of the B27 Subtype Distribution and Clinical Phenotype between JAS and AAS Groups

There was no significant difference in distribution of B27-negative patients and different B27 subtypes between JAS and AAS groups, shown in Table 1. The preliminary study of our research team has shown no significant difference in the constituent ratio of B27 subtypes between the two groups [7], and in the present study B27-negative patients were brought into this table, and there was also no significant difference in the distribution between JAS and AAS groups.

### 3.3. Comparison of Clinical Features between Different B27 Subtypes in the JAS Group

Because the data of BASFI, ESR, and CRP was not normally distributed, comparison was done after logarithmic transformation. As shown in Tables 2 and 3, there was no significant difference in the diagnostic age, male preponderance, family histories, hip arthritis, iridocyclitis, and BASFI among the four different groups in JAS patients. Peripheral arthritis, enthesitis, BASDAI, ESR, and CRP were significantly higher in patients with HLA-B^*∗*^2704 than in those with B27-negative. Enthesitis and ESR were significantly higher in patients with HLA-B^*∗*^2705 than in those with B27-negative. The Incidence of waxy digitus was more common in HLA-B^*∗*^2705 group than that in HLA-B^*∗*^2704 group. The onset age of HLA-B^*∗*^2715 group (5, 9, and 13 years) was much earlier than that in the other three groups.

### 3.4. Comparison of Clinical Features between JAS and AAS Groups

The group of JAS had higher male preponderance than AAS. And peripheral arthritis, enthesitis, and hip arthritis were more common in patients with JAS than AAS, shown in Table 4.

### 3.5. Comparison of Clinical Features in JAS and AAS Patients with HLA-B^**∗**^2704 and HLA-B^**∗**^2705

The comparison of clinical features in JAS and AAS patients with HLA-B^*∗*^2704 and HLA-B^*∗*^2705 was shown in Table 5. In patients with HLA-B^*∗*^2704, male preponderance was more obvious in JAS, and peripheral arthritis, enthesitis, and hip arthritis were also more common in JAS patients. There was no significant difference in these indicators between JAS and AAS patients with HLA-B^*∗*^2705.

## 4. Discussion

Among JAS patients with HLA-B^*∗*^2704, HLA-B^*∗*^2705, and HLA-B^*∗*^2715, there were no significant difference in male ratio, positive family history, peripheral arthritis, enthesitis, hip arthritis, iridocyclitis, and indicators, for example, BASDAI, BASFI, ESR, and CRP, which showed that the pathogenesis of different subtypes might be similar.

In the comparison between JAS patients with HLA-B27-positive and HLA-B27-negative, patients with peripheral arthritis and enthesitis in the HLA-B27-negative group were less than those in HLA-B^*∗*^2704 group; the inflammatory indicators, such as BASDAI, ESR, and CRP, in patients with HLA-B27-negative were lower than those in patients with HLA-B^*∗*^2704, which indicated the milder level of inflammation in HLA-B27-negative patients. Our previous research on a group of AAS patients showed that the onset age of patients with HLA-B27-positive was less than that in those with HLA-B27-negative [8]. Both of the two studies above showed the important role of HLA-B27 in the pathogenesis of AS. A lot of evidence illuminated that HLA-27 directly participated in the pathogenesis of AS, and the direct evidence was from the study of transgenic rats, in which human B27 gene was inserted into rats; then symptoms similar to AS appeared in the transgenic rats; and the more gene replication fragment of B27 in rats, the more obvious symptoms [9]. As the ancestor subtype, HLA-B^*∗*^2705 was most common type in Europe and America, so most basic researches including gene cloning and transgenic animals were about HLA-B^*∗*^2705 subtype. As for HLA-B^*∗*^2704 and HLA-B^*∗*^2715 subtypes, our research focused mainly on clinical aspects; further basic researches are still needed, so as to explore the pathogenesis and to find new methods of diagnosis and treatment.

The onset ages of the three patients with HLA-B^*∗*^2715 were 5, 9, and 13 years and were significantly earlier than HLA-B^*∗*^2704, HLA-B^*∗*^2705, and B27-negative group; meanwhile the duration of HLA-B^*∗*^2715 group was longer than that of HLA-B^*∗*^2705 and B27-negative groups. As a rare subtype, HLA-B^*∗*^2715 was first reported in 2001 and only found in Asia [10, 11], and there were no more than 20 cases found so far in our previous study [7, 8]; most of them were AS patients. Six sporadic cases were found in present research, and three of them were JAS patients with early onset age, indicating that this subtype was related to the disease.

There were significant difference in male ratio, peripheral arthritis, enthesitis, and hip arthritis in JAS than AAS patients with HLA-B^*∗*^2704 (shown in Table 5). The different phenotypes in juvenile and adult patients with the same subtype showed the complexity of AS pathogenesis. The study about pedigree and twins showed the multigenic mode of AS hereditary susceptibility. Hence, the difference between JAS and AAS with the same B27 subtype may be related to other genes and the different genetic expression of B27 under the influence of environment. As HLA-B^*∗*^2704 is the predominant subtype in Han population [7], the numbers of patients in HLA-B^*∗*^2705 and HLA-B^*∗*^2715 groups were small. There were no significant difference in male ratio, peripheral arthritis, enthesitis, and hip arthritis between JAS and AAS patients with HLA-B^*∗*^2705, and this was not consistent with many studies in Caucasians [12, 13]; our results should be further estimated in larger samples.

Our study showed that hip involvement was more common in JAS than AAS (shown in Tables 4 and 5); this was similar to the studies in China, Taiwan, and India [3, 6], and there was no significant difference in the B27-negative and B27-positive groups. Forty percent of patients with juvenile spondyloarthropathy progressed to functional disability in 10 to 15 years [14], and the hip involvement closely associated with poor prognosis [15]. Recent researches show that abnormality of bone loss can appear in early spondyloarthropathies and JAS; hip BMD significantly negatively correlated with BASDAI [16, 17]. These findings emphasize the need for more alertness for hip involvement at an early stage of JAS.

## 5. Conclusions

Our study showed that JAS patients with different B27 subtypes had similar features in most of manifestations, and JAS and AAS with the same subtype could have distinctive courses. Therefore, early diagnosis, hip detection, and control of systemic active inflammation will be helpful for improving the prognosis of JAS patients.

## Figures and Tables

**Table 1:** Distribution of patients with different B27 subtype and B27-negative in JAS and AAS groups.

	JAS	AAS	*P*
HLA-B27-negative	11/145 (7.59%)	41/360 (11.39%)	1.000
HLA-B^*∗*^2704	120/145 (82.76%)	275/360 (76.39%)	1.000
HLA-B^*∗*^2705	11/145 (7.59%)	38/360 (10.56%)	1.000
HLA-B^*∗*^2715	3/145 (2.07%)	3/360 (0.83%)	1.000
HLA-B^*∗*^2702	0/145 (0)	3/360 (0.83%)	0.561

**Table 2:** Comparison of clinical features, Bath AS indices, and inflammatory markers in JAS patients with B27-negative and different B27 subtypes.

HLA-B27 subtype	Age (y)	Onset age (y)	Disease duration (y)	BASDAI (score)	BASFI (score)	ESR (mm/h)	CRP (mg/L)
HLA-B27-negative	18.09 ± 3.65 (*n* = 11)	14.09 ± 2.11 (*n* = 11)	4.01 ± 4.39 (*n* = 11)	2.05 ± 1.42 (*n* = 11)	1.55 (1.21, 3.43 ) (*n* = 8)	7.00 (4.00, 9.40) (*n* = 11)	1.10 (0.60, 5.00) (*n* = 7)
HLA-B^*^2704	21.79 ± 8.02 (*n* = 120)	13.54 ± 2.31 (*n* = 120)	8.25 ± 8.06 (*n* = 120)	**4.22** ± **1.95** ^*^ (*n* = 112)	3.00 (1.90, 5.20) (*n* = 112)	**32.00 (14.00**, **48.75)** ^*^ (*n* = 120)	**18.55 (5.9**, **49.78)** ^*^ (*n* = 120)
HLA-B^*^2705	18.36 ± 3.96 (*n* = 11)	13.55 ± 1.77 (*n* = 11)	4.82 ± 4.46 (*n* = 11)	3.61 ± 1.26 (*n* = 9)	1.6 (1.00, 4.20) (*n* = 9)	**14.0 (7.00**, **48.00)** ^*^ (*n* = 11)	5.80 (1.45, 34.30) (*n* = 11)
HLA-B^*^2715	24.33 ± 6.43 (*n* = 3)	**9.00** ± **4.00** ^∗#a^ (*n* = 3)	**15.33** ± **4.16** ^∗a^ (*n* = 3)	3.28 ± 2.79 (*n* = 2)	1.125 (1.00, 1.25) (*n* = 2)	**65.00 (4.00**, **135.00)** ^*^ (*n* = 3)	15.53 (1.50, 113.45) (*n* = 3)

Note: (1) data in the table was mean ± standard deviation or median (upper quartile, lower quartile); (2) ^*^ comparison between groups of different B27 subtypes and group of B27-negative, *P* < 0.05; (3) ^#^ comparison between HLA-B^*∗*^2715 and HLA-B^*∗*^2704 group, *P* < 0.05; (4) ^a^ comparison between HLA-B^*∗*^2715 and HLA-B^*∗*^2705 group, *P* < 0.05.

**Table 3:** Comparison of clinical features in JAS patients with B27-negative and different B27 subtypes.

HLA-B27 subtype	Gender (male)	Peripheral arthritis	Enthesitis	Hip arthritis	Family histories	Waxy digitus	Iridocyclitis
HLA-B27-negative	11/11	1/11	2/11	2/11	2/11	0/11	0/11
HLA-B^*∗*^2704	112/120	**73/120** ^*^	**82/120** ^*^	49/120	34/120	1/118	6/120
HLA-B^*∗*^2705	10/11	6/11	**6/11** ^*^	4/11	3/11	**1/11** ^#^	2/11
HLA-B^*∗*^2715	3/3	2/3	2/3	0/3	2/3	0/3	0/3

Note: (1) data in the table for each index number of positive cases and total cases; (2) ^*^ comparison between groups of different B27 subtypes and group of B27-negative, *P* < 0.01; (3) ^#^ comparison between HLA-B^*∗*^2705 and HLA-B^*∗*^2704 group, *P* < 0.05.

**Table 4:** Comparison of clinical features between JAS and AAS groups.

	JAS	AAS	*P*
Gender (male)	136/145 (93.79%)	306/360 (85.00%)	**0.007**
Peripheral arthritis	82/145 (56.55%)	87/310 (28.06%)	<**0.001**
Enthesitis	92/145 (63.45%)	155/310 (50.00%)	**0.003**
Hip arthritis	55/145 (37.93%)	61/305 (20.00%)	<**0.001**
Family histories	41/145 (28.28%)	63/310 (20.32%)	0.086
Waxy digitus	2/143 (1.40%)	8/231 (3.46%)	0.230
Iridocyclitis	8/145 (5.52%)	26/310 (8.39%)	0.417

**Table 5:** Comparison of clinical features in JAS and AAS patients with HLA-B^*∗*^2704 and B^*∗*^2705.

	HLA-B^*∗*^2704			HLA-B^*∗*^2705			
	JAS	AAS	*P*1	JAS	AAS	*P*2
Gender (male)	112/120 (93.33%)	237/275 (86.18%)	**0.042**	10/11 (90.91%)	33/38 (86.84%)	0.720
Peripheral arthritis	73/120 (60.83%)	65/238 (27.31%)	<**0.001**	6/11 (54.54%)	14/37 (37.84%)	0.329
Enthesitis	82/120 (68.33%)	114/238 (47.90%)	<**0.001**	6/11 (54.54%)	24/37 (64.86%)	0.539
Hip arthritis	49/120 (40.83%)	48/241 (19.92%)	<**0.001**	4/11 (36.36%)	5/32 (15.62%)	0.303
Family histories	34/120 (28.33%)	54/238 (22.69%)	0.207	3/11 (27.27%)	7/37 (18.92%)	0.553
Waxy digitus	1/118 (0.85%)	7/177 (3.95%)	0.161	1/11 (9.09%)	0/27	0.117
Iridocyclitis	6/120 (0.05%)	21/238 (8.82%)	0.385	2/11 (18.18%)	3/37 (8.11%)	0.342
